# Clinical case study meets population cohort: identification of a *BRCA1* pathogenic founder variant in Orcadians

**DOI:** 10.1038/s41431-023-01297-w

**Published:** 2023-03-16

**Authors:** Shona M. Kerr, Emma Cowan, Lucija Klaric, Christine Bell, Dawn O’Sullivan, David Buchanan, Joseph J. Grzymski, Cristopher V. van Hout, Gannie Tzoneva, Alan R. Shuldiner, James F. Wilson, Zosia Miedzybrodzka

**Affiliations:** 1 MRC Human Genetics Unit, University of Edinburgh, Institute of Genetics and Cancer, Western General Hospital, Crewe Road, Edinburgh, EH4 2XU UK; 2 Department of Medical Genetics, Ashgrove House, NHS Grampian, Aberdeen, AB25 2ZA UK; 3 Center for Genomic Medicine, Desert Research Institute, Reno, NV USA; 4 Renown Health, Reno, NV USA; 5 Regeneron Genetics Center, Tarrytown, NY USA; 6 Laboratorio Internacional de Investigatión sobre el Genoma Humano, Campus Juriquilla de la Universidad Nacional Autónoma de México, Querétaro, Querétaro, 76230 México; 7 Centre for Global Health Research, Usher Institute, University of Edinburgh, Teviot Place, Edinburgh, EH8 9AG UK; 8 Medical Genetics Group, School of Medicine, Medical Sciences, Nutrition and Dentistry, University of Aberdeen, Polwarth Building, Aberdeen, AB25 2ZD UK

**Keywords:** Rare variants, Breast cancer

## Abstract

We multiply ascertained the *BRCA1* pathogenic missense variant c.5207T > C; p.Val1736Ala (V1736A) in clinical investigation of breast and ovarian cancer families from Orkney in the Northern Isles of Scotland, UK. We sought to investigate the frequency and clinical relevance of this variant in those of Orcadian ancestry as an exemplar of the value of population cohorts in clinical care, especially in isolated populations. Oral history and birth, marriage and death registrations indicated genealogical linkage of the clinical cases to ancestors from the Isle of Westray, Orkney. Further clinical cases were identified through targeted testing for V1736A in women of Orcadian ancestry attending National Health Service (NHS) genetic clinics for breast and ovarian cancer family risk assessments. The variant segregates with female breast and ovarian cancer in clinically ascertained cases. Separately, exome sequence data from 2088 volunteer participants with three or more Orcadian grandparents, in the ORCADES research cohort, was interrogated to estimate the population prevalence of V1736A in Orcadians. The effects of the variant were assessed using Electronic Health Record (EHR) linkage. Twenty out of 2088 ORCADES research volunteers (~1%) carry V1736A, with a common haplotype around the variant. This allele frequency is ~480-fold higher than in UK Biobank participants. Cost-effectiveness of population screening for *BRCA1* founder pathogenic variants has been demonstrated at a carrier frequency below the ~1% observed here. Thus we suggest that Orcadian women should be offered testing for the *BRCA1* V1736A founder pathogenic variant, starting with those with known Westray ancestry.

## Introduction

Pathogenic variants in *BRCA1* confer a high lifetime risk of breast and ovarian cancer [1–3]. Genetic testing for pathogenic variants in *BRCA1* and *BRCA2* is widely available in breast and ovarian cancer, to enable not only early detection and risk reduction, but also to guide cancer treatment, e.g. consideration of the use of olaparib in chemotherapy regimens [4]. Predictive testing of unaffected family members is well established, with pre-symptomatic carriers of *BRCA1* pathogenic variants being offered risk-reducing prophylactic bilateral mastectomy, bilateral salpingo-oopherectomy and annual magnetic resonance breast imaging as standard care.

In isolate populations, a pathogenic variant present in a founding or early member can become widespread in later generations, contributing significantly to the overall disease burden. Pathogenic variants in *BRCA1* and *BRCA2* have been described in several isolate and founder populations worldwide, notably Ashkenazi and Sephardi Jews, and Icelanders [5, 6]. Genetic screening programmes focused on founder variants in such genes can be cost-effective [7–10].

The Northern Isles of Scotland—the Orkney and Shetland archipelagos—have the most divergent and isolated of all British and Irish populations, with the highest degree of kinship and Norse admixture in the British Isles and Ireland, evidenced in the extensive genealogies, and genome-wide analyses [11]. Research by ourselves and others has demonstrated enrichment of rare and low frequency functional variants in isolated populations, including Orkney [12]. Enriched rare variants of large effect are of most clinical relevance.

Viking Genes (www.ed.ac.uk/viking) comprises three Northern Isles cohort studies aiming to explore genetic causes of disease—Orkney Complex Disease Study (ORCADES), VIKING I and VIKING II. ORCADES contains a rich data resource of more than 2000 deeply phenotyped and exome sequenced research subjects with three or four grandparents from the Orkney Islands, ideal for analyses of the frequency and penetrance of clinically relevant variants in the Orcadian population. UK Biobank is a large-scale cosmopolitan biomedical database containing genetic, lifestyle and health information from half a million participants in the UK [13]. Linkage to NHS routine electronic health record (EHR) data adds a longitudinal component to both the ORCADES and UK Biobank cohorts. These research cohorts, although not perfect representations of the populations from which they sought to recruit, are sufficiently unbiased for estimation of population frequency of genetic variants.

The NHS Grampian genetics team observed the *BRCA1* missense variant, c.5207T > C; p.Val1736Ala, in a number of ovarian and breast cancer cases from Orkney. The *BRCA1* c.5207T > C; p.Val1736Ala variant is a conservative amino acid substitution in the carboxyl-terminal domain, a region known to be important in BRCA1 function. In vitro studies suggested that the variant disrupts BRCA1 activity [14, 15]. Independent evidence for pathogenicity comes from saturation genome editing of *BRCA1* exons in HAP1 cells, which revealed V1736A to be non-functional in cultured cells [16]. A report was published of a severe phenotype patient with ovarian cancer at age 28, short stature, microcephaly and significant toxicity from chemotherapy, with compound heterozygous *BRCA1* variants, c.2457delC, and c.5207T > C; as well as loss of heterozygosity in associated tumours [17]. This, together with segregation data, led to reclassification of V1736A from a variant of unknown significance to likely pathogenic [17]. The interpretation that V1736A is a pathogenic variant was corroborated by an expert panel in the Clinvar database [18], accession VCV000037648, annotated as pathogenic by multiple sources. Our own co-segregation studies in the Orcadian clinical super-kindred detailed below (Methods and data available on request) support the pathogenic nature of the variant.

Here, we report for the first time the multiple ascertainment of the *BRCA1* pathogenic missense variant c.5207T > C; p.Val1736Ala (V1736A) rs45553935 as part of routine clinical care in breast and ovarian cancer families from Orkney. We then demonstrate relatedness of V1736A gene carriers using genealogy and haplotyping, and use ORCADES to estimate the population based variant frequency, consider penetrance and make the case for population screening of the variant in ancestral Orcadians. This is an exemplar of the value of phenotyped population cohorts for informing genetic health policy.

## Materials (subjects) and methods

### Clinical case note review

Women with breast and/or ovarian cancer with the *BRCA1* missense variant, c.5207T > C; p.Val1736Ala, were identified at the Orkney genetic clinic, and family history of cancer in consenting living and deceased family members was recorded and confirmed from medical records as part of routine clinical genetics care. These oral histories from multiple consultands from multiple nuclear families were supplemented with genealogical information from the Scottish Register of Births, Marriages and Deaths to link family members genealogically.

### ORCADES research volunteer recruitment

Recruitment to ORCADES took place from 2005 to 2011, through advertisement and word of mouth. Volunteers were required to be aged 18 or over, with two or more grandparents born in Orkney. More than 90% had three or four Orcadian grandparents. The response rate was excellent, with the final cohort size comprising more than 10% of the total Orkney adult population. Participants attended at least two clinics in Orkney, one for fasting venepuncture and one for physical measurements, and provided broad-ranging consent for research, including for whole genome sequencing, and for their research data to be linkable to their NHS electronic health records. Blood (or very occasionally, saliva) samples from participants were collected, processed and stored using standard operating procedures and managed through a laboratory information management system at the Edinburgh Clinical Research Facility, University of Edinburgh.

### ORCADES cohort pedigree information

Records of the births, marriages and deaths in Orkney are held at the General Register Office for Scotland (New Register House, Edinburgh). These records, along with relationship information obtained from study participants and genealogies available online, were used to construct a pedigree of ORCADES study participants using RootsMagic software (S&N Genealogy Supplies), which was then amended to reflect the genetic kinship between individuals using genotype data. The complete pedigree dates back to ~900 AD and comprises ~59,000 individuals.

### Genotyping

DNA from all ORCADES participants was used for genome-wide genotyping on the GSA BeadChip (Illumina) at the Regeneron Genetics Center. Monomorphic genotypes and genotypes with more than 2% of missingness and Hardy–Weinberg equilibrium (HWE) *p* < 10^-6^ were removed, as well as individuals with more than 3% of missingness. Details of genotyping, sample and variant quality control of UK Biobank genotyping data are described in Bycroft et al. [13].

### Sequencing

The fully quality controlled exome sequence data set was prepared at the Regeneron Genetic Centre, following the process detailed for UK Biobank by van Hout et al. [19]. Details of the quality control of whole exome sequencing on the 200,000 participants from the UK Biobank are described in Backman et al. [20]. The rs45553935 variant was validated by Sanger sequencing (further details in supplementary methods).

### Haplotype analysis

The ORCADES array genotype data were phased using Shapeit2 v2r837 [21], with the duoHMM option that uses the family-based nature of the data [22]. Prior to phasing, the array genotype data were lifted over from the genome build GRCh38 to GRCh37 using liftOver, followed by quality control against the HRC reference panel with Rayner’s HRC-1000G-check-bim (v4.2.13) script that was downloaded from https://www.well.ox.ac.uk/~wrayner/tools/. Details of phasing of UK Biobank genotyping data have been described [13]. Then, the phased genotypes were used to determine a shared haplotype around rs45553935 using the coarse and fine methods described earlier [23]. All methods were performed using R 4.0.2 (R Core Team 2020 https://www.R-project.org/). Haplotypes were defined with custom-built in-house scripts in R. Data handling was performed using data.table and tidyverse R packages. Plots were generated using ggplot2.

A single variant-based haplotype search was performed to determine the haplotype length between the different ORCADES carrier kindreds, and also with the UK Biobank carrier individuals, using a stepwise approach. Using phased genotype data, starting from the rs45553935 rare variant, one SNP at a time was added to define a haplotype. The procedure was repeated until haplotypes of two individuals (both known carriers) no longer matched, providing variant-level resolution of the haplotype length. The procedure was repeated for all pairs of individuals identified as carriers based on the exome sequencing data, both in the ORCADES and UK Biobank datasets. The shortest shared haplotype from ORCADES was then merged with the shortest shared haplotype from the UK Biobank to compare whether the haplotypes match across the 51 variants shared across genotyping chips. Two megabases of exome sequence around rs45553935 in a carrier from the Healthy Nevada Project [24] were merged with the corresponding region in a carrier from the ORCADES study. 1974 variants overlapped between the two exomes. A similar approach was then taken to assess potential haplotype sharing. Beginning with the rs45553935 variant, moving one SNP at a time, we compared the two genotypes, repeating the procedure until we came to opposing homozygotes, beyond which haplotypes cannot be shared. Identity-by-descent (IBD) analysis was performed using KING 2.1.5 [25].

### EHR data linkage in ORCADES

NHS routine datasets linked to ORCADES participants in July 2021, including the Scottish Cancer Registry SMR06, were accessed using a secure process as for the Generation Scotland cohort [26].

## Results

### Clinical ascertainment of the kindred

The rs45553935 (V1736A) variant was ascertained independently in nine diagnostic tests of breast and ovarian cancer patients. Six female obligate carriers were also identified. Fourteen V1736A carriers (eight females and six males) were identified from predictive tests in unaffected relatives of NHS patients. Five of these eight females have undergone risk reducing surgery and none have yet developed cancer. This gives a total of 23 positive NHS results in females.

### Population frequencies of the BRCA1 variant

Data from 2088 ORCADES participants (819 male and 1269 female) passed all exome sequence and genotype quality control thresholds. There are twenty heterozygous carriers of the V1736A variant in the ORCADES exome dataset, of whom seven are female. No other *BRCA1* variants reported as pathogenic or likely pathogenic in ClinVar were present in the ORCADES exome dataset, including the Scottish pathogenic founder variant 2800delAA (p.Lys894fs) [27]. None of the female carriers in ORCADES are compound heterozygotes for pathogenic or likely pathogenic exonic *BRCA1* alleles, for which there are multiple submitters and no conflicts in ClinVar, neither do they carry known pathogenic exonic variants in the cancer susceptibility genes *APC, BRCA2, RET, PALB2, MAX, TMEM127, BMPR1A, SMAD4, TP53, MLH1, MSH2, MSH6, PMS2, MEN1, MUTYH, NF2, SDHD, SDHAF2, SDHC, SDHB, PTEN, RB1, VHL* or *WT1*.

Information on allele frequencies in populations can be obtained from the Genome Aggregation Database (gnomAD) [28]. GnomAD v2.1.1, containing 125,748 exomes and 15,708 genomes from unrelated individuals, has no instances of the c.5207T>C; p.Val1736Ala variant, emphasising its rarity. Consistent with this, the variant is not observed at significant frequency in several population research cohorts including the Viking Health Study Shetland (Table 1). The DiscovEHR study [29] browser also indicates no V1736A carriers were recorded in 92,453 individuals in the Pennsylvanian MyCode population cohort [30]. In contrast, the first 200,000 exome sequences from the UK Biobank [19, 31] contain four instances (Table 1). This corresponds to a UK allele frequency of 0.00001, ~480-fold lower than we found in Orkney. None of the four UK Biobank subjects was born in the Northern Isles; two live in Scotland and two in England. Three of the four UK Biobank research participant V1736A carriers are female. One, aged in her late fifties at assessment, has ovarian cancer ICD-10 codes in the EHR dataset (Table 1). The two other female variant carriers, in their 50s and 60s at assessment, and the single male, have no reported ICD-10 codes relating to hereditary breast–ovarian cancer. Small numbers of variant carriers are also reported in two databases of genomic data from cancer cases, CanVar [32] and BCAC, the Breast Cancer Association Consortium (Table 1). However, the V1736A variant was not observed in sufficient numbers of cases and controls to allow for estimation of cancer risks in BCAC [33].

**Table 1:** Frequencies of BRCA1 p.Val1736Ala carriers in a range of genomic datasets.

Dataset	Description	Number of Genomes	Carriers of BRCA1 p.Val1736Ala variant	Cases of HBOC (females)	Cohort Publication Reference
gnomAD v2.1.1	Unrelated individuals from genetic studies	125,748 exomes and 15,708 genomes	0	-	[28]
Viking Health Study Shetland	Isolate population cohort from Shetland, UK	2106 exome sequences	0	-	[23]
DiscovEHR	Unselected population cohort in Pennsylvania, USA	92,453 exome sequences	0	-	[30]
ORCADES	Isolate population cohort from Orkney, UK	2088 exome sequences	20: 13 males and 7 females	1	[45]
UK Biobank	Cosmopolitan population cohort from UK	200,643 exome sequences	4: 1 male and 3 females	1	[31]
Healthy Nevada	Population health study from Nevada, USA	26,906 clinical ‘Exome+’ sequences	1 (female)	1	[24]
Breast Cancer Association Consortium (BCAC)	Breast cancer cases and controls, worldwide	42,671 cases with European ancestry, iCOGS custom genotyping array	2	2	[33]
CanVar-UK	Sequenced exomes of cancer patients England & Wales	28,936 total probands tested	9 (females)	9	[32]

### Origin of the V1736A variant

**Fig. 1:**
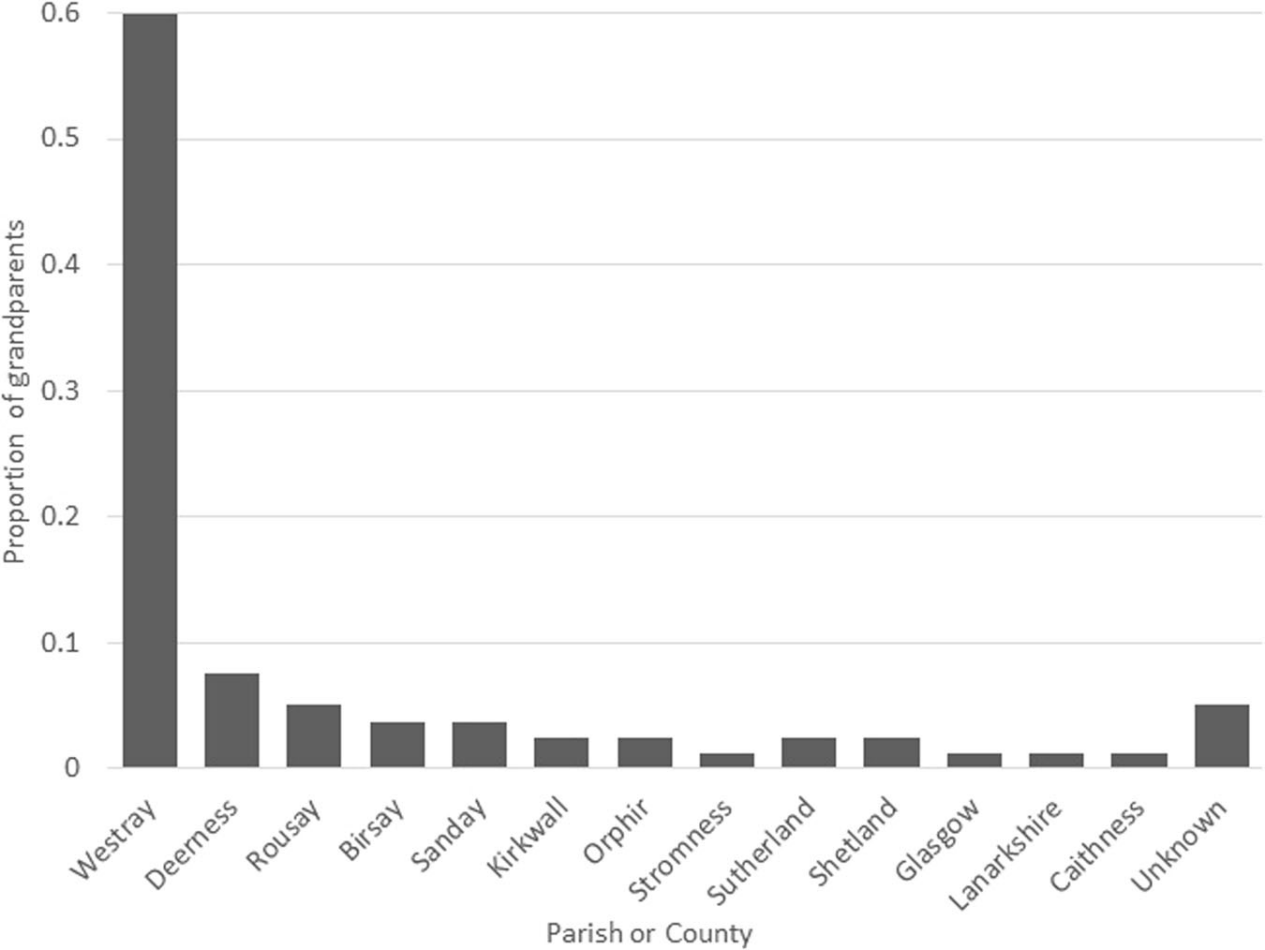
Grandparental ancestry of carriers in ORCADES. The first eight columns are parishes or isles of Orkney. The remainder are locations elsewhere in Scotland, or unknown. Of all 80 grandparents of the carriers, 60% were from Westray, with the majority of the remainder coming from other parishes or isles of Orkney.

Oral histories and data from the Scottish register of births, marriages and deaths clinical genealogy service traced the clinical cases to two lineages with founders born c. 1800, on the small island of Westray, in the North Isles of Orkney. Of the ORCADES variant carriers, eight out of twenty had four grandparents born in Westray, and all but one of them had at least one Westray grandparent. Of all 80 grandparents of the carriers, 60% were from Westray, with most of the remainder coming from other parishes or isles of Orkney (Fig. 1).

**Fig. 2:**
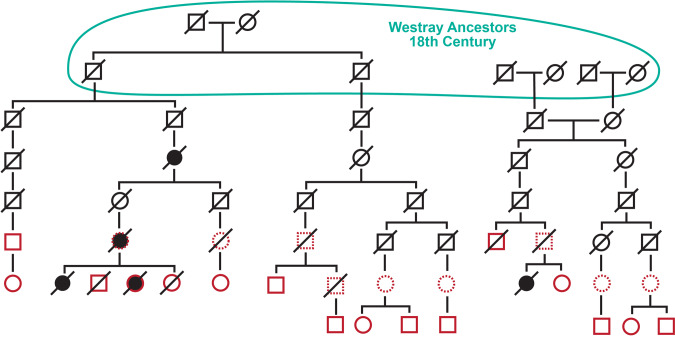
Outline pedigree of two kindreds (A and B) from the ORCADES study. Filled circles are breast or ovarian cancers, red outlines are sequenced V1736A carriers, dotted red outlines are obligate carriers. The founders of kindred A, the largest, were born in Westray in the 1760s. All four of the other kindreds also eventually lead back to Westray common ancestors, in the 19th century (but with deeper ancestry there back to the same time depth). In kindred C, mostly resident in the East Mainland of Orkney, the Westray common ancestors were born in the early 1800s.

The pathogenic variant carriers ascertained clinically and in the ORCADES study fit into five kindreds descending from five separate couples. There are four kindreds from the ORCADES, and two from the Clinical Genetics dataset, of which two overlap, giving a total of five kindreds. While most of these kindreds show distant kinship with one another, e.g. being fifth or sixth cousins, this is not uncommon among individuals with Westray heritage, and so it was not possible to be certain of the path of segregation of the variant to each of the carriers living today. Ancestral non-paternity and adoption events may have also influenced the path of segregation down the pedigree. Some of the connections are likely to date prior to *c*.1750, before which few paper genealogy records are available. Given the population structure of the island, and limited contribution of ancestors to descendants, it is likely that the kindreds which cannot be linked together genealogically do in fact connect in the preceding few generations at some point before *c*.1700, as demonstrated by their shared haplotype (see below). What is clear is that the ancestry goes back over 250 years in the island of Westray (Fig. 2).

### All V1736A carriers tested share a common haplotype

**Fig. 3:**
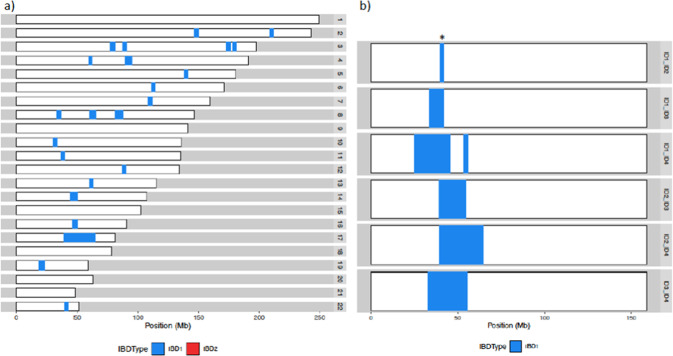
Haplotype sharing. **a** Genome-wide identity-by-descent sharing between two ORCADES carriers from different kindreds. In addition to the shared *BRCA1* haplotype on chromosome 17, background sharing due to Westray ancestry can be seen across the genome. **b** Haplotype sharing across chromosome 17 for all pairwise combinations of representatives of each of the four kindreds in ORCADES. Mb, megabase; IBD, identity-by-descent; * denotes the shortest shared haplotype.

All twenty heterozygous V1736A carriers in ORCADES share the same haplotype at the variant locus, with a minimum length of ~2 Mb (Fig. 3). Access was given to exome sequence data surrounding the same pathogenic variant in a breast cancer patient (Table 1) described by Grzymski et al. [24], for comparative haplotype analysis. Analysis of exome data from this carrier participant in the Healthy Nevada Project [24] revealed that there were no opposing homozygote genotypes versus an ORCADES carrier across 676 SNPs, totalling 407 kb, consistent with them sharing one haplotype identical-by-descent in this region.

Analysis of haplotypes in the genotype data from the four UK Biobank participants carrying the variant showed that they all shared a ~1.1 Mb long haplotype, which was identical to the Westray haplotype from ORCADES. DNA was not available for haplotype analysis from the family described in Domchek et al. [17].

### Penetrance of V1736A in Orcadians

In addition to the exome sequence information, and the detailed study data collected in the recruitment clinics, linkage to routine NHS data in the EHR provides a longitudinal component to many research cohorts, including ORCADES and the UK Biobank. The morbidity (hospital admissions, SMR01) and cancer registry (SMR06) datasets are particularly useful for research on people living in Scotland. These data have been obtained for almost all participants in the ORCADES cohort.

The mean age of the seven female carriers at time of recruitment to ORCADES (baseline) was 54, and six gave permission for EHR linkage. Two V1736A carrier participants died over 80 years of age, one of whom had ovarian cancer recorded as cause of death. None of the remaining carriers had a diagnosis of breast or ovarian cancer recorded, and four with EHR linkage survive (Table 2). Nine female obligate carriers linking two branches of the ORCADES pedigrees together were also ascertained (Table 2). Research study family history questionnaires reported that two died of breast cancer, three died of other causes and three remained cancer-free (for one there was no information). Scottish death registration certificates reveal that the great-grandmother of four of the ORCADES carriers died of breast cancer in the mid-1930s, and their close relative who died of breast cancer in her late fifties.

**Table 2:** Cancer status of women from Orkney carrying BRCA1 p.Val1736Ala.

Carrier Identification Route	Total *n* (Female)	Breast or Ovarian Cancer (*n*)	Age of Case(s) at Diagnosis (years)	Deaths (n)	Age of living cancer-free females
NHS Diagnostic or Obligate Carrier	15	13	Min 25–29 Max 75–79 Mean = 55.7	15	N/A
NHS Predictive Carrier	8	0 (Prophylactic surgery chosen by 5)	N/A	0	Min 15–19 Max 85–89 Mean = 48.9
ORCADES Research Cohort	7	1	70 s	2	Min 55–59 Max 70–74 Mean = 61
ORCADES Obligate Carrier (Relative of research participant)	9	2	50 s	5	70–74 Mean = 73

Together with the clinically-ascertained cases, we have thus identified a total of 37 women of Orcadian heritage with the variant, only two of whom overlap between the clinically-ascertained cases and ORCADES/obligate carriers. Importantly, comparison of common ancestors between the clinically ascertained and population-based pedigrees demonstrated that it is likely that only two out of the seven female variant carriers in the ORCADES population cohort have already been offered genetic testing as part of the cascade testing of the index family.

## Discussion

Our combined approach of a family-based case study and systematic analysis within a population cohort has identified the pathogenic variant p.Val1736Ala *BRCA1* in 1% of Orcadians. All carriers share a novel rare long haplotype background. The variant is likely to have arisen in a founder individual from Westray, Orkney, at least 250 years ago. Individuals not ascertained by cascade testing of relatives from the clinical pedigree have been identified by the cohort study. In a previous work on the rare *KCNH2* variant, p.Gly584Ser [23], which causes long QT syndrome, a form of familial cardiac arrythmia, we similarly identified carriers in our Northern Isles research populations who could not have been ascertained from oral history based cascade testing. Indeed, for both actionable variants, up to seventh degree affected relatives were ascertained. This highlights the value of research cohorts in describing the burden of rare clinical variants, which may in turn help planning genetic services.

Although it possible that individuals with a family history of disease might be more likely to participate in genetic research studies, in ORCADES bias with respect to the breast or ovarian cancer phenotypes is mitigated by the large size of the cohort as a proportion of the Orcadian population. Furthermore, the recruitment information referred to “common diseases such as heart disease, eye disease, stroke and diabetes” and not cancers specifically.

Pathogenic variants in actionable genes like *BRCA1* are often considered to be more penetrant in the clinical context of a family history of the relevant condition than in population-based cohorts, due to co-inheritance of multiple lower penetrance modifiers, and ascertainment bias contributes to risk over-estimation [34]. However, the penetrance of the predominant Ashkenazi pathogenic sequence variants is demonstrated as largely related to the variants themselves, with minor contribution of the specific family history [35]. In the clinical genetics setting, *BRCA1* penetrance to 80 years of 79.5% (95%CI 75.5–83.5%) for breast cancer and 65% (95%CI 75–84%) for ovarian cancer are reported [36], whereas population cohorts indicate lower risks. For example, reported penetrance of pathogenic/loss-of-function variants in *BRCA1* in population cohorts is heterogeneous (mean 38%, range 0–100%) [34] and influenced by family history [37]. Despite the large size of the kindred we report here, power is limited to precisely estimate the penetrance of V1736A. The available data on number of cases and age of onset suggest more modest breast cancer penetrance than is typically seen in genetic clinic families with *BRCA1*, which fits with a missense variant with some residual function. However, the penetrance data we present is similar to that of many other *BRCA1* pathogenic variants [2, 36].

Breast cancer risk in women from breast-ovarian cancer families born before 1940 is considerably less than in those born after [36]. This is likely not only due to reduced longevity, but also to lower body mass, higher parity, prolonged breast feeding and dietary factors in earlier generations. This observation fits with our data (available on request) that indicate higher ovarian than breast cancer risk. Counselling in the family has highlighted this familial context. Most women with positive predictive tests in the family have chosen breast MRI screening and risk-reducing bilateral salpingo-oopherectomy by age 40. Uptake of risk reducing mastectomy has been limited, but in line with wider local experience. To date, none of those undergoing predictive testing for the variant have developed breast or ovarian cancer. VIKING II, which is recruiting those of Northern Isles ancestry regardless of domicile [38], has highlighted scientifically for the first time the extent of Orcadian emigration, across Canada, New Zealand, and Australia but also in England and mainland Scotland.

High penetrance *BRCA1* and *BRCA2* founder pathogenic variants are described in several populations such as Iceland, the Ashkenazim, Poland, Norway and others, and testing for these is well established [5, 6, 8, 9]. For example, in the French-Canadian founder population, twenty variants in *BRCA1*, *BRCA2*, and *PALB2* that predispose families to breast and ovarian cancer have been identified at increased frequencies. A recent paper demonstrated that genetic screening in that population could identify up to 10% of those who currently present with early-onset breast and ovarian cancer, prior to a diagnosis [39]. However, the challenges of likely reduced penetrance in those without a known family history of cancer, and cost, have limited adoption of asymptomatic BRCA screening outwith selected founder populations in resource-limited healthcare systems. The carrier frequency of 1% that we observe for the c.5207T>C; p.Val1736Ala *BRCA1* variant in the Orkney population is higher than some of the founder variants reported in these populations, and cost effectiveness of population-based screening for *BRCA1* founder pathogenic variant at 1% frequency has been reported in Sephardi Jewish women [10]. Recently, NHS England announced its first programme of targeted founder BRCA pathogenic variant screening for people with at least one Jewish grandparent (NHS to launch expanded BRCA genetic testing for Jewish community - The Jewish Chronicle (thejc.com)). In support of this approach, an economic evaluation of population-based *BRCA1*/*BRCA2* pathogenic variant testing across multiple countries and health systems has recently been published [40].

Although to date, the consent framework of most research cohorts does not allow the return of results about carrier status of actionable variants [19], participants in clinical and biobanking studies often wish to receive their results, particularly about “actionable” findings. This has recently stimulated publication an international policy for returning genomic research results [41]. Others also recommend the return of results following detection of hereditary breast and ovarian cancer risk to adult population-based biobank participants [41–43]. Our ongoing recruitment to a new Northern Isles cohort study, VIKING II, offers new and existing cohort members the option of consent to return of selected clinically actionable results, and the return of this variant will be prioritised in that process. Relevant participants resident in Scotland will be offered clinically accredited verification on a new sample by the NHS clinical genetics service.

We propose that all women of Orcadian ancestry (worldwide) with a diagnosis of breast cancer should be offered a targeted test for this variant, if a *BRCA1/BRCA2* gene screen is not offered as part of their clinical care. This targeted test for Orcadians with a family history of breast or ovarian cancer is now routine practice in the NHS Grampian clinic, but we know this approach will miss many of those at risk.

Slightly over 11,000 females live in Orkney, of whom ~9300 are adult, and ~70% of residents have two or more Orcadian ancestors. To date, we have identified less than half of the resident Orcadian V1736A carriers. We are therefore preparing a business case for population-based screening for the variant through primary care community hubs in Orkney, using the inexpensive Sanger sequencing assay that is established in the NHS Grampian genomics laboratory. We propose to pilot this programme by offering a test to Westray residents of known Westray ancestry.

High penetrance genes contribute only a proportion of genetic cancer risk, and V1736A is only one of many contributors to breast and ovarian cancer risk in Orcadians. Common low penetrance variants identified through genome-wide association studies explain a further component. Polygenic risk scores (PRS) are being considered for enhancement of risk stratification, both in the general population and in BRCA1/2 carrier populations [44]. Genetic drift of common low penetrance variants may limit the portability of scores developed elsewhere. Work is ongoing to assess the utility of PRS in Orcadians, and to determine if low penetrance breast cancer-associated SNPs are enhanced or reduced in this population. We are also examining in detail the clinical utility of testing for other Northern Isles drifted pathogenic variants identified through clinical practice and the Viking Genes studies.

In conclusion, we propose that women with two or more Orcadian grandparents should be offered testing for the V1736A variant, regardless of family history of breast or ovarian cancer. The analyses presented here of the *BRCA1* variant are relevant beyond the modern population of Orkney, both as an exemplar and due to emigration to elsewhere in the British Isles and the New World. Future research will explore further genetically drifted loci observed as part of clinical care in Orkney and Shetland in the Viking Genes research cohorts.

## Supplementary information

Supplementary methods

## Data Availability

Some information (e.g. age and nature of a diagnosis) could potentially make individuals identifiable, so has not been shown, or is presented in aggregate form. These data can be made available to legitimate researchers affiliated to an academic organisation through application to the corresponding author. There is neither Research Ethics Committee approval, nor consent from ORCADES participants, to permit open release of the individual level research data underlying this study. The datasets generated and analysed during the current study are therefore not publicly available. Instead, the research data and/or DNA samples are available from accessQTL@ed.ac.uk on reasonable request, following approval by the Data Access Committee and in line with the consent given by participants.
