# Supplementary material for: Clinical case study meets population cohort: identification of a BRCA1 pathogenic founder variant in Orcadians
Source: Eur J Hum Genet. 2023 Mar 16;31(5):588–95. doi: 10.1038/s41431-023-01297-w (PMC10172333; doi:10.1038/s41431-023-01297-w)
Supplement: Supplementary file 1 — Supplementary methods [file 41431_2023_1297_MOESM1_ESM.docx]

**Supplementary methods**

***Sequencing***

*Exome sequencing*

Briefly, the multiplexed samples were sequenced on the Illumina NovaSeq 6000 platform using S2 flow cells. The raw sequencing data was then processed by automated analysis on the DNAnexus platform^20^ where files were converted to FASTQ format, followed by alignment to GRCh38 genome reference using the BWA-mem^21^. Duplicated reads were identified and flagged using the Picard tool (<http://picard.sourceforge.net> 2018). Genotypes for each individual sample were called using the WeCall variant caller (<https://github.com/Genomicsplc/wecall> 2018).

Out of 2,131 sequenced samples, the following were removed: 33 samples that were identified as duplicates, 3 whose genetically-determined sex was discordant with the reported sex, 4 with high rates of heterozygosity/contamination, 2 with low sequence coverage (less than 80% of targeted bases achieving 20X coverage) and 1 discordant with genotyping chip. A PVCF file containing all samples was then created using the GLnexus joint genotyping tool^22^. The “Goldilocks” dataset was created by filtering out genotypes with read depth less than 7 reads, keeping only variants that had at least one heterozygous variant genotype with allele balance ratio greater than or equal to 15% (AB ≥ 0.15), or at least one homozygous variant genotype. This was followed by additional filtering of all variants with more than 10% of missingness and HWE p<10^-6^. Details of the quality control of whole exome sequencing on the 200,000 participants from the UK Biobank are described in Backman *et al^23^*.

*Sanger sequencing*

The rs45553935 variant was validated Sanger sequencing. The primers 5′AGCTAAGATCTGAACCCGAGA3′ and 5′CCACCACGCCCAACTAATTT3′ were designed using Primer3 software (Thermo Fisher Scientific) and used to generate a fragment of 586 base pairs for analysis. All 20 heterozygous variant calls from the ORCADES exome dataset were independently verified using this method.
